# Comparative Transcriptome Analysis Revealed Genes Commonly Responsive to Varied Nitrate Stress in Leaves of Tibetan Hulless Barley

**DOI:** 10.3389/fpls.2016.01067

**Published:** 2016-07-21

**Authors:** Zexiu Wei, Xingquan Zeng, Cheng Qin, Yulin Wang, Lijun Bai, Qijun Xu, Hongjun Yuan, Yawei Tang, Tashi Nyima

**Affiliations:** ^1^Tibet Academy of Agricultural and Animal Husbandry SciencesLhasa, China; ^2^State Key Laboratory of Barley and Yak Genetic Resources and ImprovementLhasa, China; ^3^Institute of Agricultural Resources and Environment Science, Tibet Academy of Agricultural and Animal Husbandry SciencesLhasa, China; ^4^Agricultural Research Institute, Tibet Academy of Agricultural and Animal Husbandry SciencesLhasa, China; ^5^Zunyi Academy of Agricultural SciencesZunyi, China; ^6^Best Biological Technology Co., LTDChengdu, China

**Keywords:** Tibetan hulless barley, nitrogen stress, comparative transcriptomics, differential gene expression, transcription factors

## Abstract

Nitrogen (N) deprivation or excess can lead to dramatic phenotype change, disrupt important biological processes, and ultimately limit plant productivity. To explore genes in Tibetan hulless barley responsive to varied N stress, we utilized a comparative transcriptomics method to investigate gene expression patterns under three nitrate treatments. The transcriptome of the control (optimal-nitrate, ON) sample was compared with that of free-nitrate (FN), low-nitrate (LN), and high-nitrate (HN) treatment samples, identifying 2428, 1274, and 1861 genes, respectively, that exhibited significant differences in transcript abundance. Among these, 9 genes encoding ribulose bisphosphate carboxylases exhibited up-regulated expression under varied N stress. We further compared FN vs. ON and LN vs. ON to investigate the impact of stress degree on gene expression. With the aggravation of stress, more genes were differentially expressed and thus possibly involved in the response to nitrogen deficiency. Cluster and functional enrichment analysis indicated that the differentially expressed genes (DEGs) in FN were highly enriched in response to stress, defense response, and gene expression regulation. Comprehensive comparison analysis further suggested that Tibetan hulless barley could respond to varied N stress by regulating multiple common biological processes and pathways such as nitrogen metabolism, carbon metabolism, and photosynthesis. A large number of specific DEGs involved in diverse biological processes were also detected, implying differences in the potential regulatory patterns of low- and high-N stress response. Notably, we also identified some NIN-like proteins and other transcription factors significantly modulated by these stresses, suggesting the involvement of these transcription factors in N stress response. To our knowledge, this study is the first investigation of the Tibetan hulless barley transcriptome under N stress. The identified N-stress-related genes may provide resources for genetic improvement and promote N use efficiency.

## Introduction

Nitrogen (N), a necessary factor for life, plays crucial roles in plant growth and development, and represents the essential constituent of most macromolecules and many secondary and signaling compounds such as proteins, nucleic acids, and hormones (Krapp, 2015). Plants need to acquire N efficiently from the soil for growth, especially under conditions of highly fluctuating N availability, and develop a sophisticated uptake system to cope with N fluctuation in the soil and maintain normal growth and development (Wang et al., 2012).

N has both positive and negative effects on plant development and growth. Numerous studies have shown that N deprivation can lead to dramatic changes and even disrupt important biological processes in plants such as N metabolism and photosynthesis (Lu et al., 2001; Zhao et al., 2005). N deprivation can decrease leaf area index, plant height, and shoot weight, and ultimately limits plant productivity (McCullough et al., 1994; Pandey et al., 2000). Conversely, increasing evidence demonstrates that excess N can also negatively affect plant growth (Tian et al., 2008; Saiz-Fernández et al., 2015). Significant negative effects of high N doses include the restriction of root growth, leaf expansion, and whole plant development (Zhang et al., 1999; Saiz-Fernández et al., 2015). In addition, changes in plant hormone levels were shown to play a relevant role in this inhibitory effect (Kiba et al., 2011; Saiz-Fernández et al., 2015).

The associated responsive genes and mechanisms of N stress response have attracted much attention during the last decades and the underlying regulatory mechanisms appeared to have been completely elucidated based on extensive studies in model plants such as *Arabidopsis* and maize (Wang et al., 2012). Another important crop, Tibetan hulless barley (*Hordeum vulgare* L. var. *nudum* Hook. f.), is widely cultivated at higher altitudes on the Tibet Plateau (Baik and Ullrich, 2008). However, gene regulation and signaling pathways related to N stress response in this plant are only partly understood. Recently, the Tibetan hulless barley genome was sequenced as reported in our previous study (Zeng et al., 2015); this data is expected to provide a framework for the identification and functional characterization of genes important from a global perspective for the improvement of Tibetan hulless barley and for basic research. In the current study, in order to gain a better understanding of the N stress response mechanism, we generated a series of transcriptome datasets to explore the transcriptional changes of hulless barley leaves under varied N conditions and analyze the differences among free nitrate, low nitrate and high nitrate stress responses using comparative transcriptome analysis. The results will likely facilitate further discovery of N stress-responsive genes and provide an important foundation for future studies on the cloning and functional characterization of these genes in hulless barley.

## Materials and methods

### Sample preparation

For comparative transcriptome analysis, we utilized the Tibetan hulless barley cultivar Zangqing 320, which shows a tolerance to N stress. The seeds were sterilized in 10% (v/v) H_2_O_2_ for 15 min, rinsed with distilled water, and germinated in a plant growth chamber under a 14 h day/10 h night cycle (28°C/21°C day/night temperature cycle) with 60–70% relative humidity. The uniform seedlings with three leaves were transferred into Hoagland solution. After 2 weeks, seedlings of uniform size and growth were picked randomly and planted into four treatment groups including optimal-nitrate (ON) (4 mM/L nitrate), free-nitrate (FN) (0 mM/L nitrate), low-nitrate (LN) (0.04 mM/L nitrate), and high-nitrate (HN) (40 mM/L nitrate) treatment groups. Ca(NO_3_)_2_·4H_2_O and NH_4_NO_3_ were used as the N source and the consequent Ca^2+^ deficiency was supplemented with CaCl_2_. The other components of the nutrient solution were as described previously (Xu et al., 2011). After being subjected to N stress for 2 days, seedling leaves were harvested and two independent replicates were collected for each sample. Plant materials were frozen in liquid nitrogen immediately and stored at −80°C until subsequent analyses.

### RNA extraction, library construction, and sequencing

Total RNA was extracted using TRIzol reagent (Invitrogen, Carlsbad, CA, USA) according to the manufacturer's protocols. Library construction was performed by staff at the Beijing Genome Institute (BGI, Shenzhen, China) comprising the following steps: enrichment of mRNA, fragment interruption, addition of adapters, size selection, and polymerase chain reaction (PCR) amplification. In total, eight paired-end libraries were constructed and 90 bp paired-end reads were generated using Illumina HiSeq™ 2000.

### Read preprocessing and identification of differentially expressed genes (DEGs)

To ensure the high quality of sequencing data, clean reads with high quality were obtained by removing low-quality sequencing reads. Gene expression quantification was conducted using RSEM software (Li and Dewey, 2011). Fragments per kilobase pair of exon model per million fragments mapped (FPKM) was used to normalize gene expression values. The pairwise comparisons between N-treated and control samples were executed using the NOIseq method (Tarazona et al., 2012). DEGs were obtained based on a threshold of significance as *P*≥0.8. Cluster analysis of expression patterns was conducted using R language and Mev v4.7.4 software (Saeed et al., 2003). Gene ontology (GO) annotation and enrichment analyses were performed based on the GO Database (http://www.geneontology.org/) involving three hierarchies: biological process, molecular function, and cellular component. Pathway enrichment analysis of DEGs was performed utilizing the KEGG database (http://www.genome.jp/kegg/).

### Quantitative real-time PCR (qPCR)

To validate the findings of the RNA-Seq assay, 20 DEGs were randomly chosen and their relative expression confirmed by qPCR analysis using the fluorescent intercalating dye SYBRGreen in the Opticon 2 detection system (MJ Research, Waltham, MA, USA). Details of the selected genes and the respective primers are listed in Table S1. Gene expression levels were normalized against the hulless barley gene *HvADP* (Ferdous et al., 2015) and calculated using the 2^−ΔΔCT^ method (Livak and Schmittgen, 2001). Three technical replicates were generated for each biological sample.

## Results

### Transcriptome sequencing and data analyses

After removing sequencing adaptors and low quality data, we obtained 50,781,646 and 51,951,054 paired-end 90 bp reads from N treated and control samples, respectively, corresponding to approximately 4.57 and 4.68 Gb data (Table S2). The results of data quality assessment showed that clean reads exhibited good quality scores with Q20 percentages of all samples over 95%, whereas the uncalled base (“*N*”) percentages were lower than 0.01% (Table S2). The GC contents were almost identical for all eight hulless barley leaf tissues, ranging from 51.43 to 54.41%. These results indicated that the read number and quality were sufficient for further analysis.

On average, we mapped 77.61% of the clean reads to the full gene set of Tibetan hulless barley (Table S3). A total of 28,481 known expressed genes were detected in all samples, out of which 21,547 genes were expressed in all samples (Table S3). Gene expression data showed a Pearson's correlation between biological replicates of over 99.08% for all samples analyzed, indicating high correlation between biological replicates (Figure S1).

### Gene expression profiles under varied N supplies in hulless barley

Compared with the ON group, 2428, 1274, and 1861 genes were classified as DEGs in the FN, LN, and HN groups, respectively (Table S4). Under FN stress, 837 genes showed increased expression and 1591 genes showed decreased expression (Table S4). In comparison, relatively fewer DEGs were identified under LN stress, wherein 675 transcripts were up-regulated and 599 were down-regulated (Table S4). In the HN group, 729 genes were induced and 1132 genes were suppressed (Table S4). Venn-diagram analysis revealed that 809 DEGs displayed differential expression under all three N stress treatments (Figure S2). In addition, 879, 138, and 407 DEGs were specifically detected in the FN, LN, and HN groups, indicating different transcriptional changes under varied N stress. To validate the accuracy and reproducibility of the RNA-Seq results, 20 DEGs were randomly chosen and the expression profiles were evaluated using qPCR. The qPCR and RNA-Seq analyses showed a positive correlation coefficient (*R*^2^ > 0.80), suggesting the reliability of the RNA-Seq results (Figure S3).

**Figure 1:**
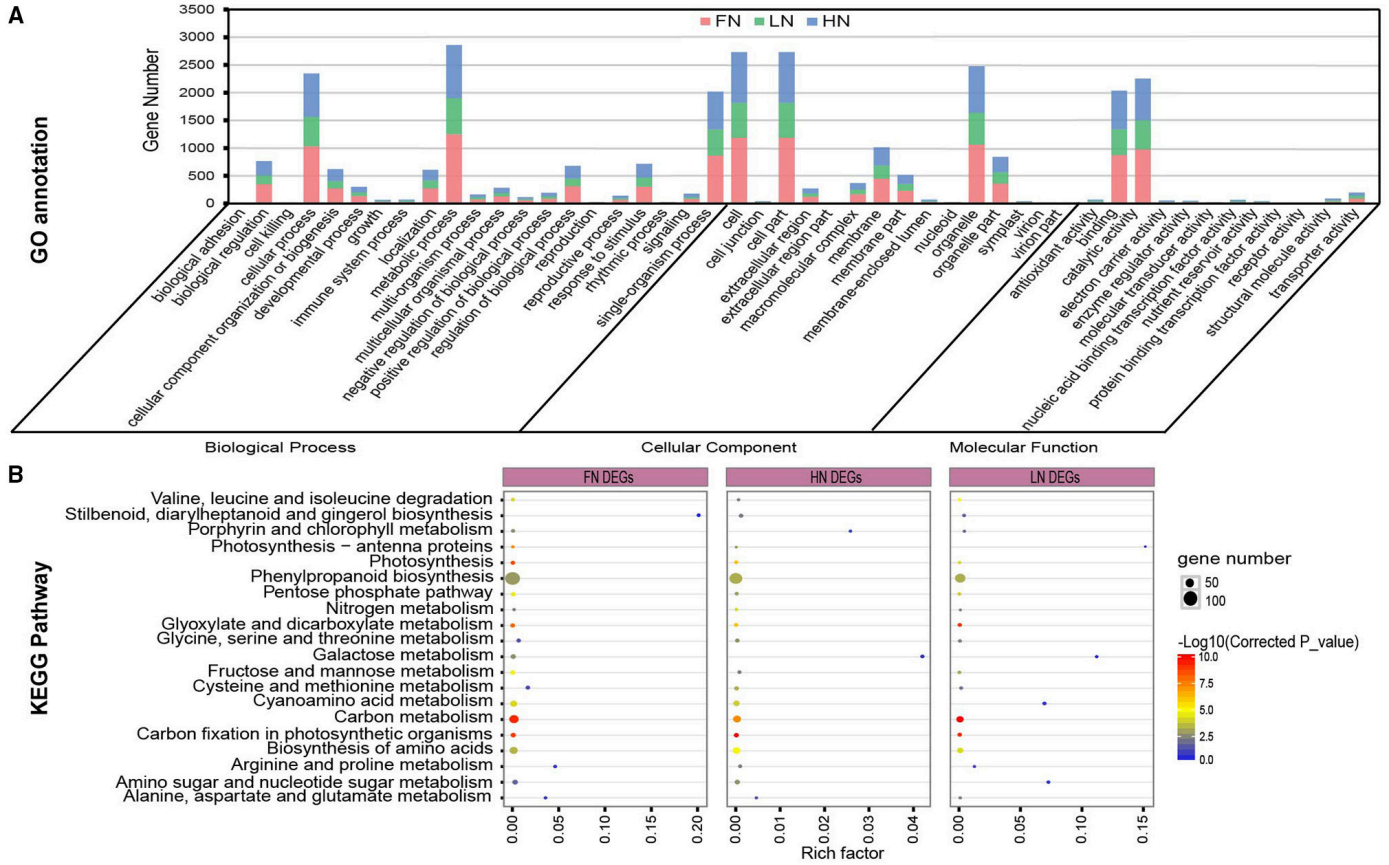
**Functional annotation and enrichment analysis of differentially expressed genes (DEGs) responsive to varied N stress**. **(A)** Gene annotation of DEGs. **(B)** KEGG enrichment analysis for DEGs.

Next, the three DEG sets were assigned to 51 GO classes by GO annotation analysis (Figure 1A). In the molecular function classification, “binding” and “catalytic activity” were dominant among the GO terms (Figure 1A). Under the classification of biological processes, “metabolic process,” “cellular process,” and “biological regulation” were prominently represented (Figure 1A). It is noteworthy that 303, 167, and 247 DEGs in the FN, LN, and HN groups, respectively, were separately annotated to “response to stimulus” (Figure 1A). The GO enrichment analyses further identified common and specific GO enrichment terms in the three DEG sets (Table S5). The DEGs in FN were significantly overrepresented in “photosystem I” (GO:0009522), “photosynthesis” (GO:0015979), and “nitrogen compound metabolic process” (GO:0006807). The DEGs in LN were found to be enriched in “positive regulation of catalytic activity” (GO:0043085), “cell killing” (GO:0001906), and “terpenoid biosynthetic process” (GO:0016114) (Table S5). In addition, the DEGs in HN were specifically enriched in “branched-chain amino acid metabolic process” (GO:0009081), “regulation of transport” (GO:0051049), and “oxidoreductase activity, acting on other nitrogenous compounds as donors” (GO:0016661) (Table S5).

KEGG pathway enrichment analysis also revealed that the three DEG sets were commonly enriched in nitrogen metabolism, N assimilation, and related metabolic pathways such as “biosynthesis of amino acids,” “carbon metabolism,” “carbon fixation in photosynthetic organisms,” “photosynthesis,” and “valine, leucine, and isoleucine degradation” (Figure 1B, Table S6).

Overall, the differences of enrichment degree and specific enrichment pathways implied that responsive differences existed between the three N stress groups. Thus, these results suggested that these pathways and processes might participate in N stress response.

### Transcriptional changes under different degrees of N starvation

To investigate the impact of stress degree differences on gene expression, we further compared the differences of DGEs between two pairwise comparisons (FN vs. ON and LN vs. ON). A total of 1010 DEGs overlapped, out of which 473 were upregulated and 368 were down-regulated (Figure S4). In addition, 254 DEGs appeared only in LN vs. ON comparison, while 1408 DEGs were in FN vs. ON comparison (Figure S4).

Hierarchical cluster analysis was then carried out based on expression profile of these common DEGs. On this basis, GO enrichment analysis of each cluster was performed and illustrated in Figure S5A. Enriched GO terms of gradually up-regulated genes under N deficiency included nitrogen compound metabolic process (GO:0006807), photosynthesis (GO:0015979), and carbohydrate catabolic process (GO:0016052). The common DEGs with down-regulated expression trend were significantly enriched in cellular amino acid catabolic process (GO:0009063), alpha-amino acid catabolic process (GO:1901606), and branched-chain amino acid catabolic process (GO:0009083). KEGG pathway enrichment revealed that numerous DEGs participated in biosynthesis of amino acids, photosynthesis, and carbon metabolism (Figure S5B). Among them, a high proportion of DEGs showed up-regulated expression profiles. The down-regulated DEGs mainly related to valine, leucine and isoleucine degradation, fatty acid degradation, and alanine, aspartate and glutamate metabolism.

**Figure 2:**
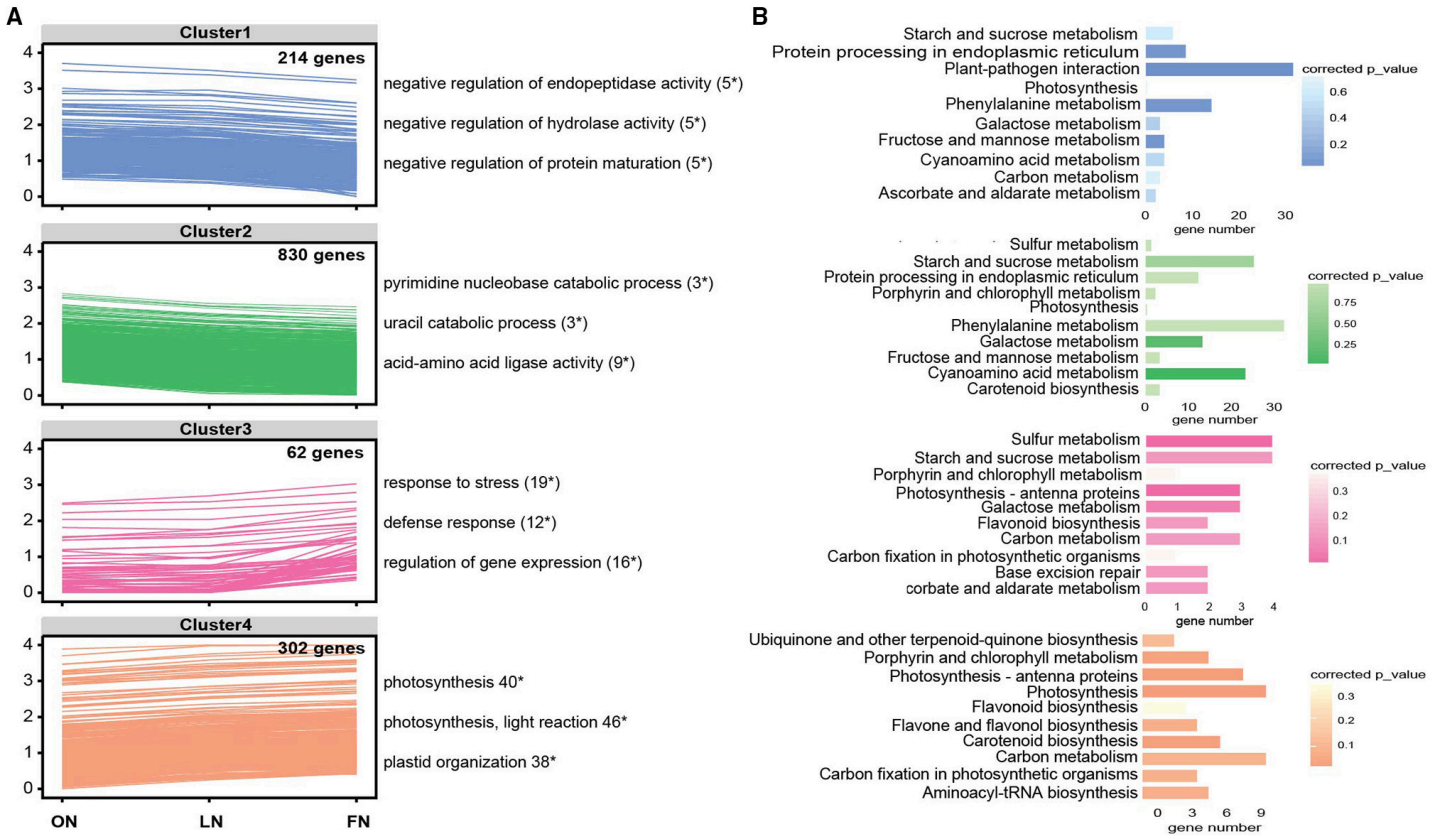
**Cluster analysis and functional analysis of specific differentially expressed genes (DEGs) in the free- (FN) vs. optimal- (ON) nitrate comparison**. **(A)** Clustering of specific DEGs based on the expression profiles (FPKM values were log10-transformed). The top GO terms and corresponding DEG number are shown on the right side; “*” represents significant enrichment (corrected *p* < 0.05). **(B)** KEGG pathway enrichment analysis of specific DEGs in the FN vs. ON comparison. The y-axis corresponds to the pathway and the x-axis shows the DEG number. The color of the dot represents the enrichment factor.

We then focused on specific DEGs in FN vs. ON comparison, and identified 4 clusters based on dynamic expression profiles using a k-means clustering approach (Li et al., 2010). Cluster 1 and 3 displayed dramatically changes under free nitrogen stress (Figure 2A). The DEGs in cluster 1 significantly overrepresented in negative regulation of endopeptidase activity (GO:0010951), negative regulation of hydrolase activity (GO:0051346), negative regulation of protein maturation (GO:1903318,). Cluster 3 were highly enriched in response to stress (GO:0006950), defense response (GO:0006952), and regulation of gene expression (GO:0010468). KEGG pathway analysis further demonstrated distinct functional enrichments in biological process among four clusters (Figure 2B). Interestingly, there were 11 DEGs in cluster 4 significantly induced expressions under free nitrogen stress, involving in photosynthesis pathway. Carbon fixation in photosynthetic organisms and carbon metabolism also belonged to enrichment pathway for cluster 4.

### Comparative gene expression analysis under N deficiency and excess conditions

**Figure 3:**
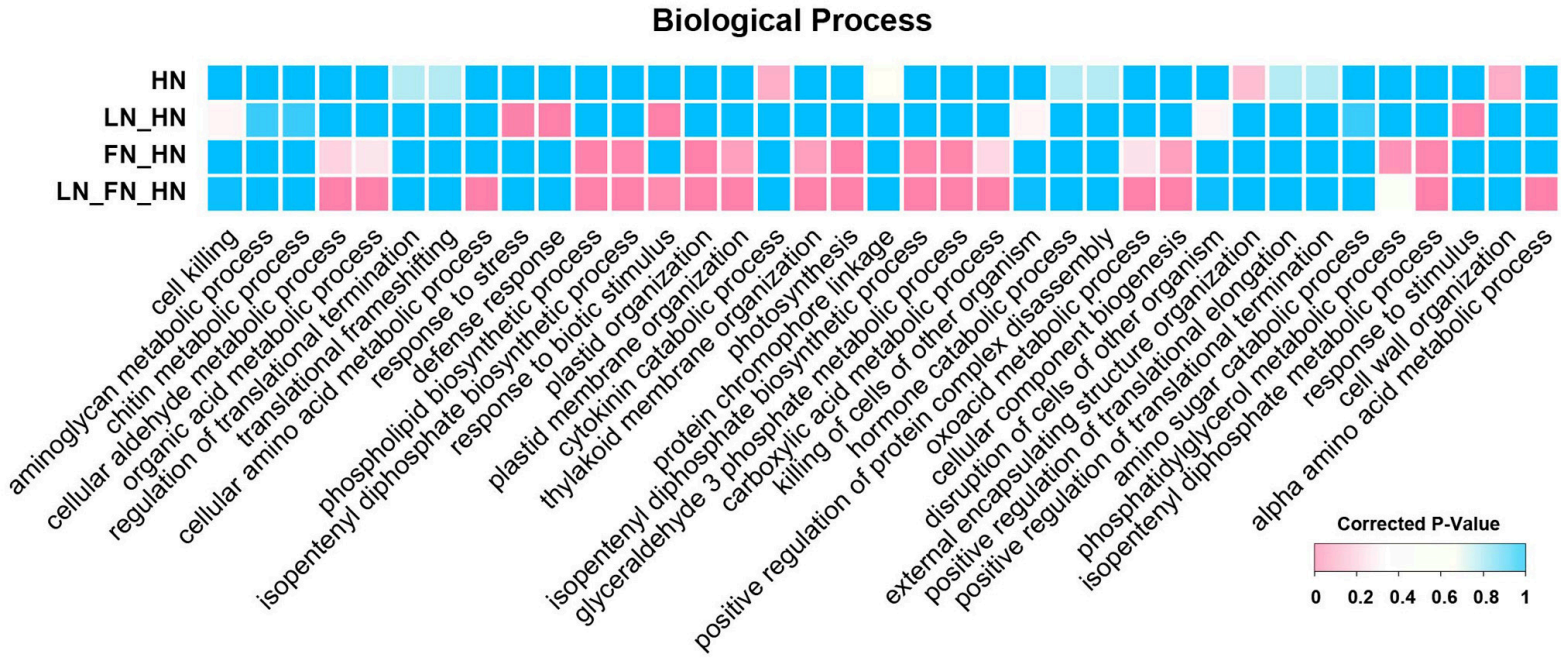
**Cross-comparison of functional enrichment analysis of DEGs in response to N stress**. Different colors in the block represent the different significance levels of the overrepresentation.

To explore the genes associates with low- and high-N stress response in Tibetan hulless barley, we performed comprehensive comparisons of the three N stress groups. We identified 809 common DEGs among the three N stress-treated groups as compared to the ON group that were associated with multiple biological processes including “cellular aldehyde metabolic process,” “cellular amino acid metabolic process,” and “photosynthesis” (Figure 3). The common DEGs were further subjected to KEGG pathway enrichment analysis, which revealed that these common DEGs were significantly enriched in “nitrogen metabolism,” “carbon metabolism,” “photosynthesis,” “biosynthesis of amino acids,” and “starch and sucrose metabolism” (Figure S6). In contrast, the 407 specific DEGs detected in the HN vs. ON comparison were not significantly enriched in any biological processes (Figure S6). However, KEGG analysis found that these specific DEGs were primarily involved in “phenylalanine metabolism,” “phenylpropanoid biosynthesis,” “cyanoamino acid metabolism,” and “mRNA surveillance pathway” (Figure S6). These results suggested that Tibetan hulless barley had the ability to respond to N stress by regulating multiple common biological processes and pathways. In addition, a large number of specific DEGs involved in diverse biological processes were detected in the HN vs. ON comparison, indicating that the regulatory patterns of low- and high-N stress response also exhibited differences.

## Discussion

Nitrogen is an essential macronutrient that directly affects agricultural productivity; accordingly, both low and high N supply has been suggested to alter plant growth and development. In this study, we utilized transcriptome sequencing to identify DEGs in hulless barley under varied N stress conditions. GO annotation and KEGG pathway analysis revealed that the three DEG sets were commonly enriched in multiple GO terms and pathways such as “nitrogen metabolism,” “biosynthesis of amino acids,” “carbon metabolism,”, “carbon fixation in photosynthetic organisms,” and “photosynthesis.” These results not only indicated that these pathways were related to N stress response but also showed that some components were simultaneously involved in three N stress response processes. Furthermore, the existence of specific DEGs and enrichment pathways for each tested group implied that different response mechanisms also existed under varied N stresses.

We then adopted a comparative trancriptomics approach to explore common transcriptional changes in response to N deficiency stresses (LN and FN). More DEGs (2428) were identified in the FN vs. ON comparison than in the LN vs. ON comparison (1274), suggesting that more DEGs were induced or suppressed with the aggravation of N deficiency stress.

**Figure 4:**
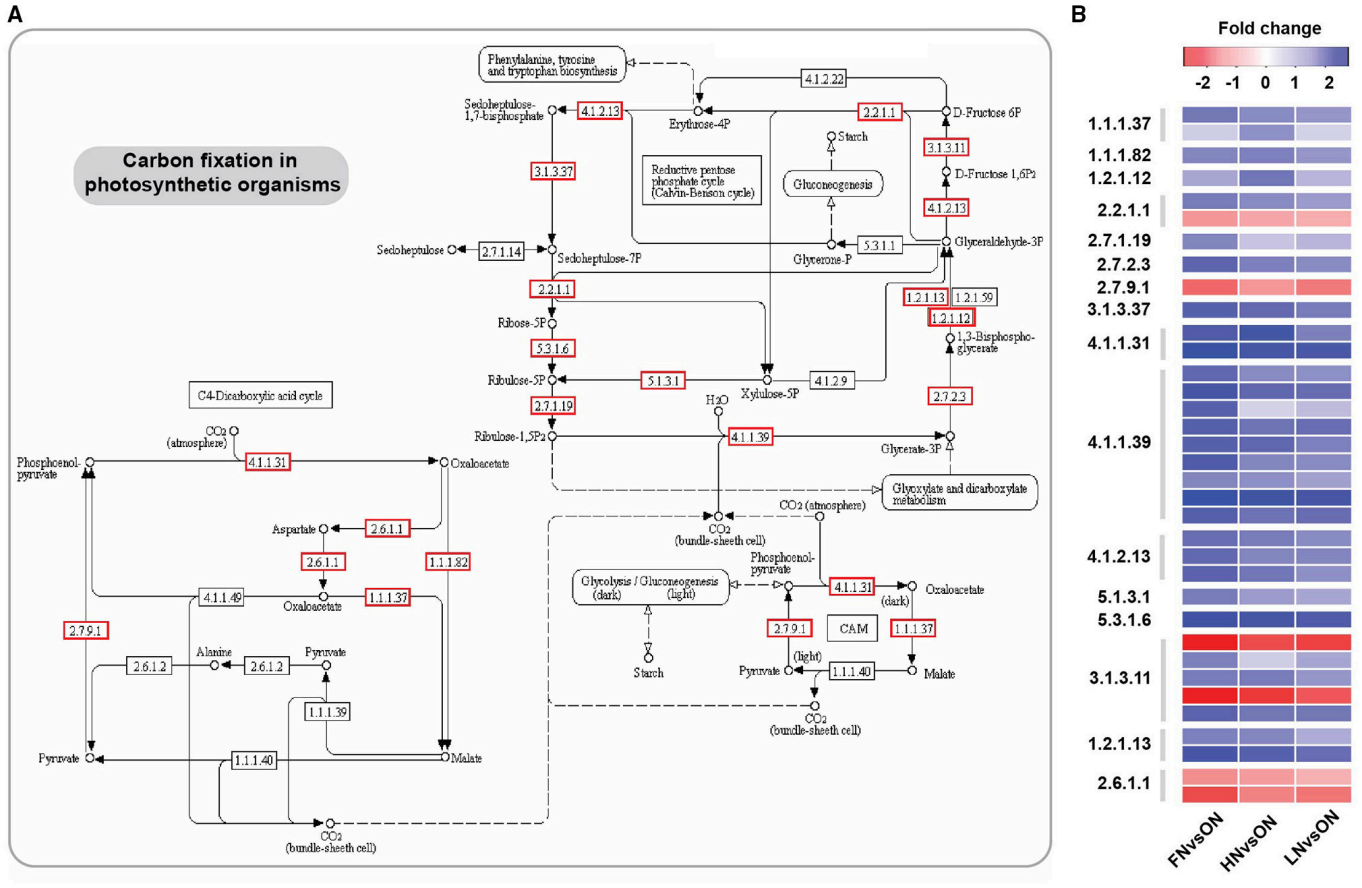
**Differentially expressed genes (DEGs) participating in carbon fixation in photosynthetic organisms under varied N treatments**. **(A)** The pathway of carbon fixation in photosynthetic organisms. Red panes represent the DEGs. **(B)** The expression pattern of DEGs involved in carbon fixation in photosynthetic organisms.

Comprehensive comparison of transcriptional changes under N deficiency and excess conditions revealed that 809 common genes were differentially expressed under all three N stresses; notably, these also were associated with “nitrogen metabolism,” “carbon metabolism,” and “photosynthesis.” These results indicated that N stress exerted significant influences on these processes. It has long been recognized that N assimilation is intrinsically linked to both photosynthetic activity and the overall carbon “C” status in plants (Nunes-Nesi et al., 2010; Krapp, 2015). In plants, the energy and C skeletons produced by photosynthesis are essential for N assimilation (Masclaux-Daubresse et al., 2010; Nunes-Nesi et al., 2010; Xu et al., 2012), and N stress has previously been found to trigger differential expression of genes involved in carbon metabolism and photosynthesis (Hakeem et al., 2012). In present study, numerous DEGs were identified in both processes. Specifically, 35 DEGs were identified to be associated with carbon fixation in photosynthetic organisms. Of these, 9 genes encoding Rubisco (ribulose bisphosphate carboxylase, EC:4.1.1.39) demonstrated up-regulated expression under varied N stress (Figure 4). Rubisco represents the rate-limiting enzyme in photosynthetic carbon fixation (Makino et al., 1985). Phosphoenolpyruvate carboxylase (*PEPC*, EC:4.1.1.31) and malate dehydrogenase (*MDH*, EC:1.1.1.37), which are involved in malate formation in root nodules (Soussi et al., 1998; Jin et al., 2015; Nazir et al., 2016), also were induced or repressed under N stress. Malate is the main substrate for supporting nitrogenase activity (Soussi et al., 1998). Overall, four genes encoding PEPC and MDH were induced by varied N stresses in the present study (Figure 4). Aspartate aminotransferase (AST, EC:2.6.1.1) is an enzyme involved in amino acids synthesis, which plays an important role in regulating carbon and nitrogen metabolism in almost all organisms (Zhou et al., 2009). Over-expression of *AST* genes in rice resulted in alteration of N metabolism and an increase of amino acid content (Zhou et al., 2009). We found that two *AST* genes were repressed under the three N stress conditions in this study (Figure 4). In addition, other genes related to carbon fixation also showed significant changes in expression abundance, such as glyceraldehyde-3-phosphate dehydrogenase (EC:1.2.1.13), fructose-1,6-bisphosphatase I (EC:3.1.3.11), and fructose-bisphosphate aldolase (EC:4.1.2.13) (Figure 4). Effective nitrogen uptake is dependent on ammonium transporters and nitrate transporters. The nitrate transporter *Hvulgare_GLEAN_10010589* represented a common DEG that showed dramatic expression changes under all three N stress conditions. Other ammonium transporters and nitrate transporters (*Hvulgare_GLEAN_10047276, Hvulgare_GLEAN_10029666*, and *Hvulgare_GLEAN_10002591*) presented differential expression only in specific N stress conditions. These N transporters still require further investigation to determine their function in N uptake, assimilation, translocation, recycling, and remobilization in hulless barley. Overall, these results suggested that Tibetan hulless barley could respond to N stress by regulating multiple common biological processes and pathways such as N metabolism, carbon metabolism and photosynthesis. Additionally, 407 specific DEGs involved in diverse biological processes were detected in HN, indicating the differences between the regulatory patterns in N deficiency and excess stress response.

In a previous study, transcription factors were demonstrated to function as molecular players involved in the dynamic regulation of gene expression in response to N stress (He et al., 2016). NIN-like proteins (NLPs) have been reported as master regulators involved in N stress responses by promoting the expression of N stress responsive genes through interaction with N response elements (Konishi and Yanagisawa, 2013; Marchive et al., 2013; Krapp et al., 2014). In particular, a chromatin immunoprecipitation-chip analysis identified that genes involved in N metabolism, the oxidative pentose phosphate pathway, sulfur and carbon metabolism, and transcription factors represented target genes of NLP7 (Marchive et al., 2013). In the current study, two genes (*Hvulgare_GLEAN_10013289* and *Hvulgare_GLEAN_10041837*), belonging to the NLP transcription factor family, exhibited significant down-regulated expression under HN stress. However, no differentially expressed *NLP* genes were detected under FN or LN stress, suggesting that differences in transcriptional changes exist under conditions of N deficiency vs. N excess.

Other previously characterized transcription factors such as MADS (Zhang and Forde, 1998), Lim-domain binding (LDB) (Rubin et al., 2009), and bZIP (Lillo, 2008) have also been shown to be involved in the regulation of N stress responses. In the present study, MADS (4 genes), LDB (2), and bZIP (3) transcription factors were also identified as participating in N stress response (Table 1). By RNA gel blot analysis, one myeloblastosis (MYB) transcription factor was found to respond to N stress in *Arabidopsis*, which showed modest induction at low nitrate concentration but good induction following high nitrate content (Wang et al., 2000). Here, we also found 9 genes encoding MYB transcription factors were differentially expressed under varied N stress. In *Arabidopsis*, the LBD transcription factors negatively regulate N-responsive genes including key genes required for N uptake and assimilation (Liu et al., 2015). We also found two genes coding LBD transcription factors (*Hvulgare_GLEAN_10001286* and *Hvulgare_GLEAN_10002368*) that showed opposite regulation patterns in the LN and FN groups. In addition, we found that the genes encoding AP2-EREBP, ARF, bHLH, C2C2-Dof, MYB-related, NAC, and WRKY were identified as being differentially expressed as well (Table 1). These transcription factors might participate in diverse biological processes and play distinct roles in N stress response.

**Table 1:** **Transcription factors with significantly different expression (|fold_change|>2) responding to varied N stress in Tibetan hulless barley**.

**Gene ID**	**Transcription factor family**	**Comparison group**	**Fold change**	**Direction of regulation**
*Hvulgare_GLEAN_10049376*	NAC	FN vs. ON	8.49	Up
*Hvulgare_GLEAN_10027452*	C2C2−GATA	FN vs. ON	8.22	Up
*Hvulgare_GLEAN_10019819*	ABI3VP1	FN vs. ON	2.64	Up
*Hvulgare_GLEAN_10043562*	Trihelix	FN vs. ON	2.52	Up
*Hvulgare_GLEAN_10005179*	ARF	FN vs. ON	2.43	Up
*Hvulgare_GLEAN_10031590*	GeBP	FN vs. ON	2.33	Up
*Hvulgare_GLEAN_10018881*	bHLH	FN vs. ON	2.17	Up
*Hvulgare_GLEAN_10003720*	GRF	FN vs. ON	2.16	Up
*Hvulgare_GLEAN_10048094*	C3H	FN vs. ON	−2.02	Down
*Hvulgare_GLEAN_10036483*	NAC	FN vs. ON	−2.04	Down
*Hvulgare_GLEAN_10003788*	ARR−B	FN vs. ON	−2.19	Down
*Hvulgare_GLEAN_10022973*	MADS	FN vs. ON	−2.19	Down
*Hvulgare_GLEAN_10031527*	C3H	FN vs. ON	−2.24	Down
*Hvulgare_GLEAN_10007185*	Trihelix	FN vs. ON	−2.26	Down
*Hvulgare_GLEAN_10041450*	bZIP	FN vs. ON	−2.28	Down
*Hvulgare_GLEAN_10008574*	WRKY	FN vs. ON	−2.35	Down
*Hvulgare_GLEAN_10037071*	bHLH	FN vs. ON	−2.44	Down
*Hvulgare_GLEAN_10055366*	NAC	FN vs. ON	−2.5	Down
*Hvulgare_GLEAN_10011348*	bHLH	FN vs. ON	−2.52	Down
*Hvulgare_GLEAN_10008615*	WRKY	FN vs. ON	−2.61	Down
*Hvulgare_GLEAN_10016772*	WRKY	FN vs. ON	−2.67	Down
*Hvulgare_GLEAN_10051673*	NAC	FN vs. ON	−3.18	Down
*Hvulgare_GLEAN_10057860*	MYB	FN vs. ON	−3.41	Down
*Hvulgare_GLEAN_10014040*	AP2−EREBP	FN vs. ON	−3.48	Down
*Hvulgare_GLEAN_10046988*	NAC	FN vs. ON	−4.74	Down
*Hvulgare_GLEAN_10013893*	NAC	FN vs. ON	−5.55	Down
*Hvulgare_GLEAN_10049706*	NAC	HN vs. ON	3.87	Up
*Hvulgare_GLEAN_10022522*	ARF	HN vs. ON	3.56	Up
*Hvulgare_GLEAN_10019819*	MYB	HN vs. ON	2.77	Up
*Hvulgare_GLEAN_10007928*	MYB−related	HN vs. ON	2.4	Up
*Hvulgare_GLEAN_10000629*	GRF	HN vs. ON	2.34	Up
*Hvulgare_GLEAN_10022750*	ARF	HN vs. ON	2.17	Up
*Hvulgare_GLEAN_10035135*	MYB	HN vs. ON	2.14	Up
*Hvulgare_GLEAN_10039025*	C2C2−GATA	HN vs. ON	2.09	Up
*Hvulgare_GLEAN_10038699*	MYB−related	HN vs. ON	−2.6	Down
*Hvulgare_GLEAN_10030584*	MYB−related	HN vs. ON	−2.6	Down
*Hvulgare_GLEAN_10059391*	C3H	HN vs. ON	−2.7	Down
*Hvulgare_GLEAN_10013289*	RWP−RK	HN vs. ON	−4.61	Down
*Hvulgare_GLEAN_10045490*	ARF	HN vs. ON	−5.23	Down
*Hvulgare_GLEAN_10002439*	NAC	LN vs. ON	2.97	Up
*Hvulgare_GLEAN_10019910*	C3H	LN vs. ON	2.96	Up
*Hvulgare_GLEAN_10026489*	TAZ	LN vs. ON	2.65	Up
*Hvulgare_GLEAN_10024972*	Tify	LN vs. ON	2.25	Up
*Hvulgare_GLEAN_10009714*	bHLH	LN vs. ON	2.04	Up
*Hvulgare_GLEAN_10011816*	GeBP	LN vs. ON	−2.06	Down
*Hvulgare_GLEAN_10015806*	WRKY	LN vs. ON	−2.22	Down
*Hvulgare_GLEAN_10033470*	MADS	LN vs. ON	−2.46	Down

Together, the findings of this study will likely identify some N-stress-related genes by comparative transcriptome analysis, and provide gene resources for genetic improvement and promote nitrogen use efficiency.

## Author contributions

Conceived and designed the experiments: ZW, XZ, CQ, and TN. Performed the experiments: ZW, XZ, YW, QW, HY, and YT. Analyzed the data: ZW, XZ, LB, and CQ. Wrote the paper: ZW, CQ, and TN. All authors have read and approved the manuscript.

### Conflict of interest statement

The authors declare that the research was conducted in the absence of any commercial or financial relationships that could be construed as a potential conflict of interest.

## Supplementary material

Figure S1 **Pearson's correlation analysis of the RNA-Seq data**.

Figure S2 **Venn diagrams of differentially expressed genes (DEGs) in response to varied N stress**.

Figure S3 **Correlation between RNA-Seq and qPCR data**. Each RNA-Seq expression data result was plotted against that from qPCR and fitted into a linear regression. Both x- and y-axes are shown in a log2 scale and each color represents a different gene.

Figure S4 **Common differentially expressed genes (DEGs) and their expression profile between free- (FN) vs. optimal- (ON) nitrate and low-nitrate (LN) vs. ON comparisons**.

Figure S5 **Functional annotation and enrichment analysis of common differentially expressed genes (DEGs) between free- (FN) vs. optimal- (ON) nitrate and low-nitrate (LN) vs. ON comparisons. (A)** Clustering and heat maps of common DEGs based on the expression profiles. **(B)** KEGG pathway analysis of common DEGs.

Figure S6 **Cross-comparison of pathway annotation of differentially expressed genes (DEGs) in response to N stress**.

Table S1 **Primers for the qPCR assays of the eight RNA-Seq libraries used in this study**.

Table S2 **Summary of sequencing data quality for Tibetan hulless barley**.

Table S3 **Summary of mapping rate and gene expression statistics based on the RNA-Seq data**.

Table S4 **Differentially expressed genes (DEGs) in response to N stress in Tibetan hulless barley**.

Table S5 **GO annotation of differentially expressed genes in response to N stress in Tibetan hulless barley**.

Table S6 **KEGG analysis of differentially expressed genes in Tibetan hulless barley responsive to N stress**.
