# Supplementary figures and images for: Comparative Transcriptome Analysis Revealed Genes Commonly Responsive to Varied Nitrate Stress in Leaves of Tibetan Hulless Barley
Source: Front Plant Sci. 2016 Jul 21;7:1067. doi: 10.3389/fpls.2016.01067 (PMC4954818; doi:10.3389/fpls.2016.01067)

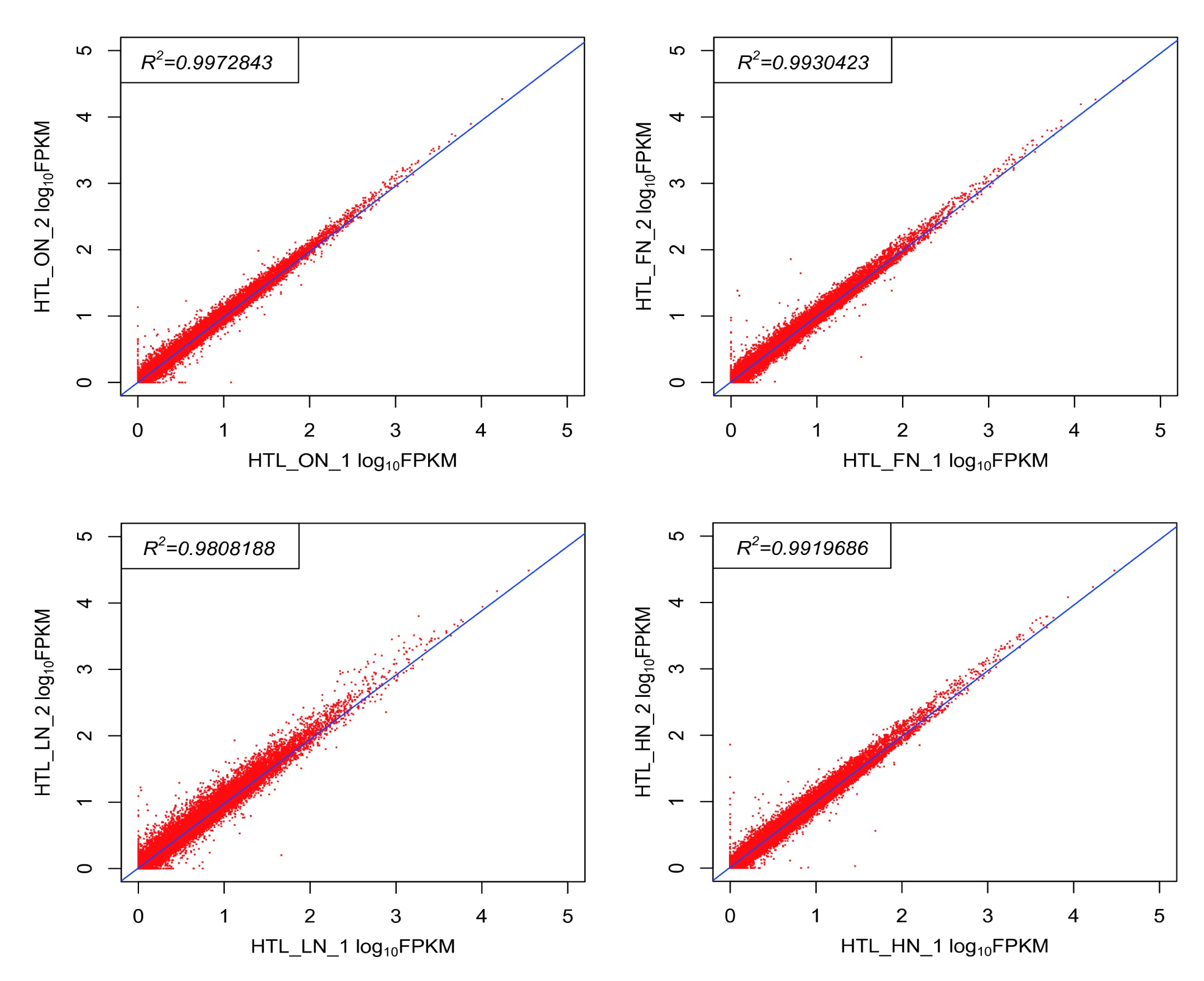

Supplement: Figure S1 — Pearson's correlation analysis of the RNA-Seq data. [file Image1.JPEG]

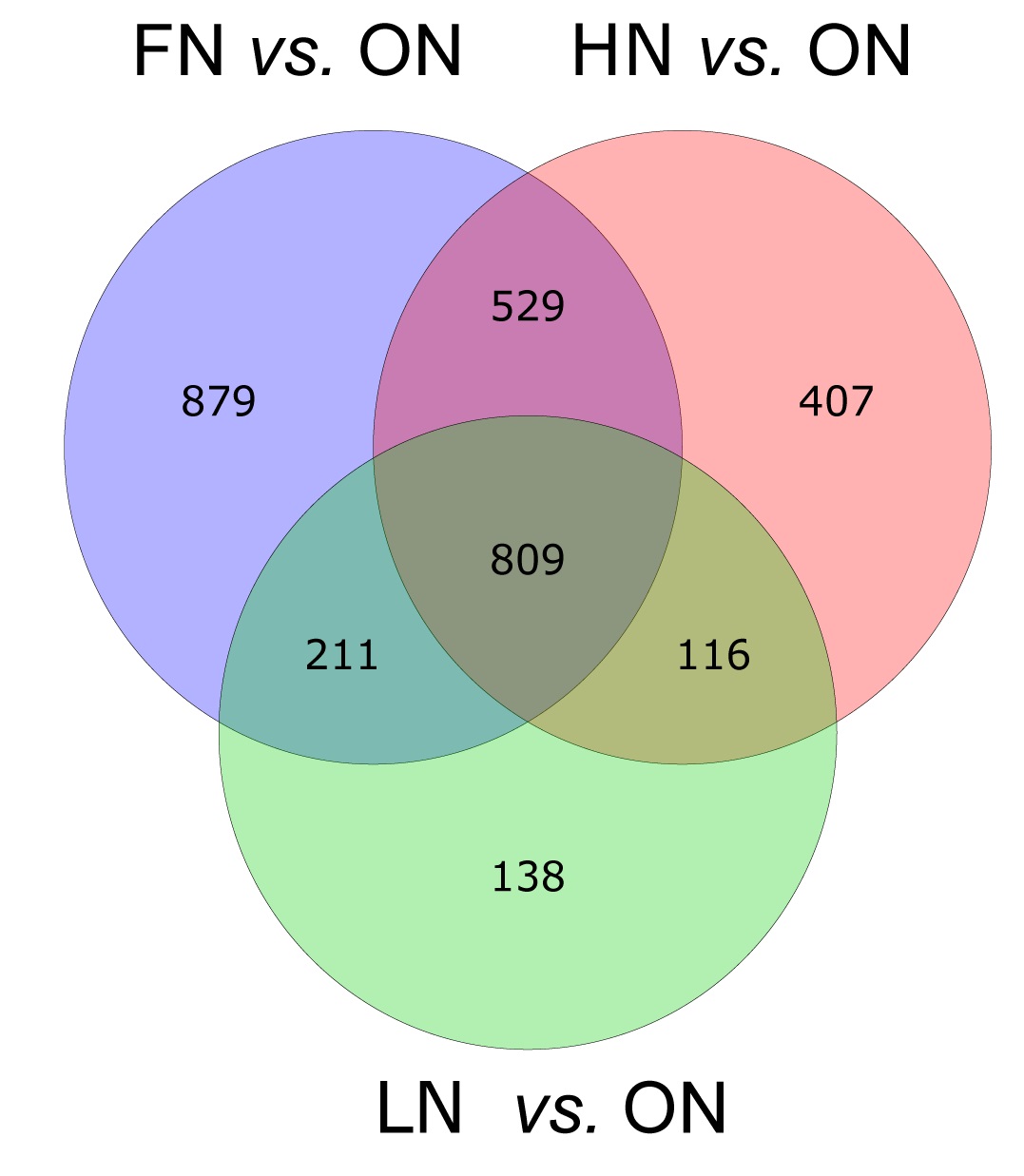

Supplement: Figure S2 — Venn diagrams of differentially expressed genes (DEGs) in response to varied N stress. [file Image2.JPEG]

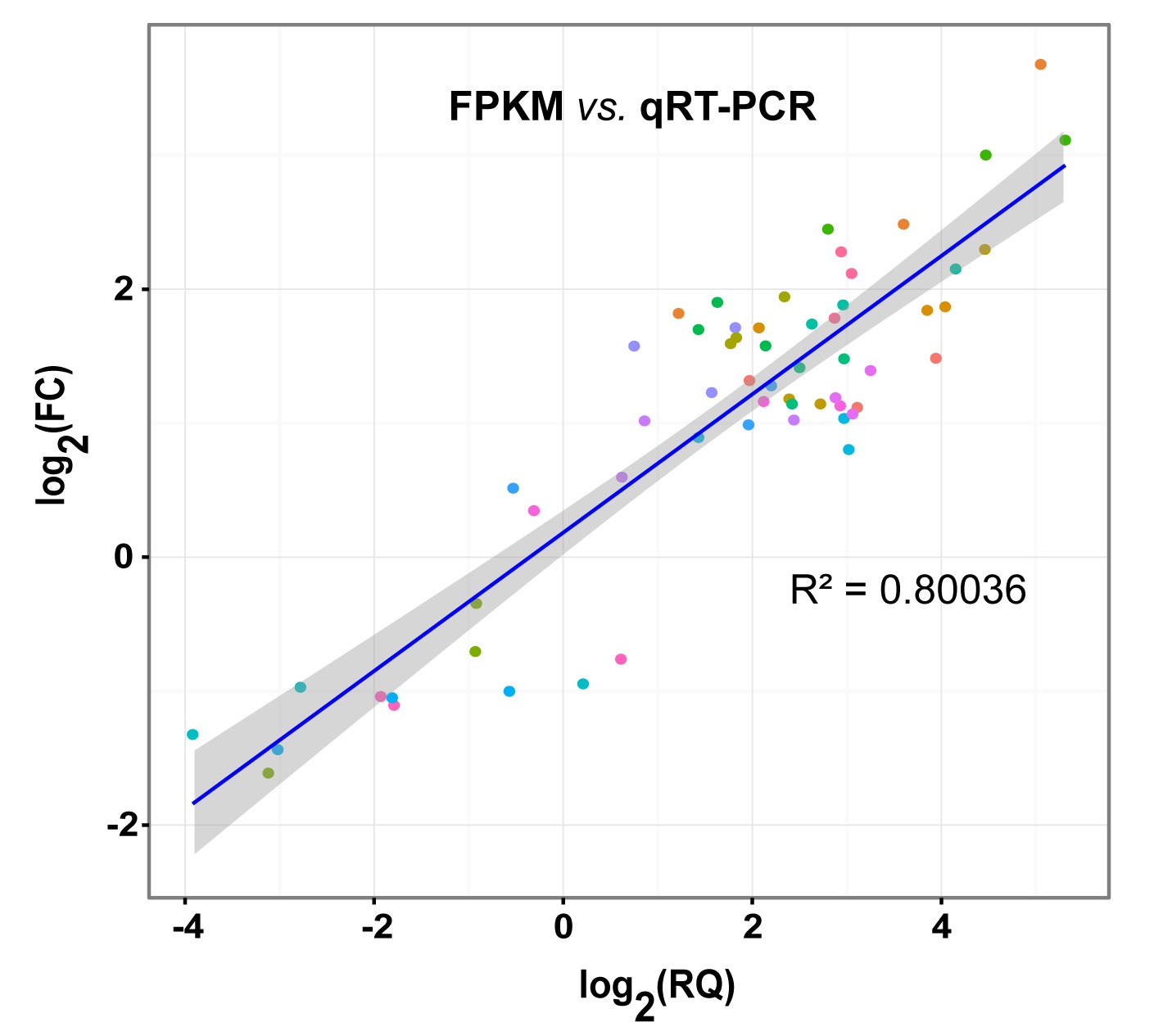

Supplement: Figure S3 — Correlation between RNA-Seq and qPCR data. Each RNA-Seq expression data result was plotted against that from qPCR and fitted into a linear regression. Both x- and y-axes are shown in a log2 scale and each color represents a different gene. [file Image3.JPEG]

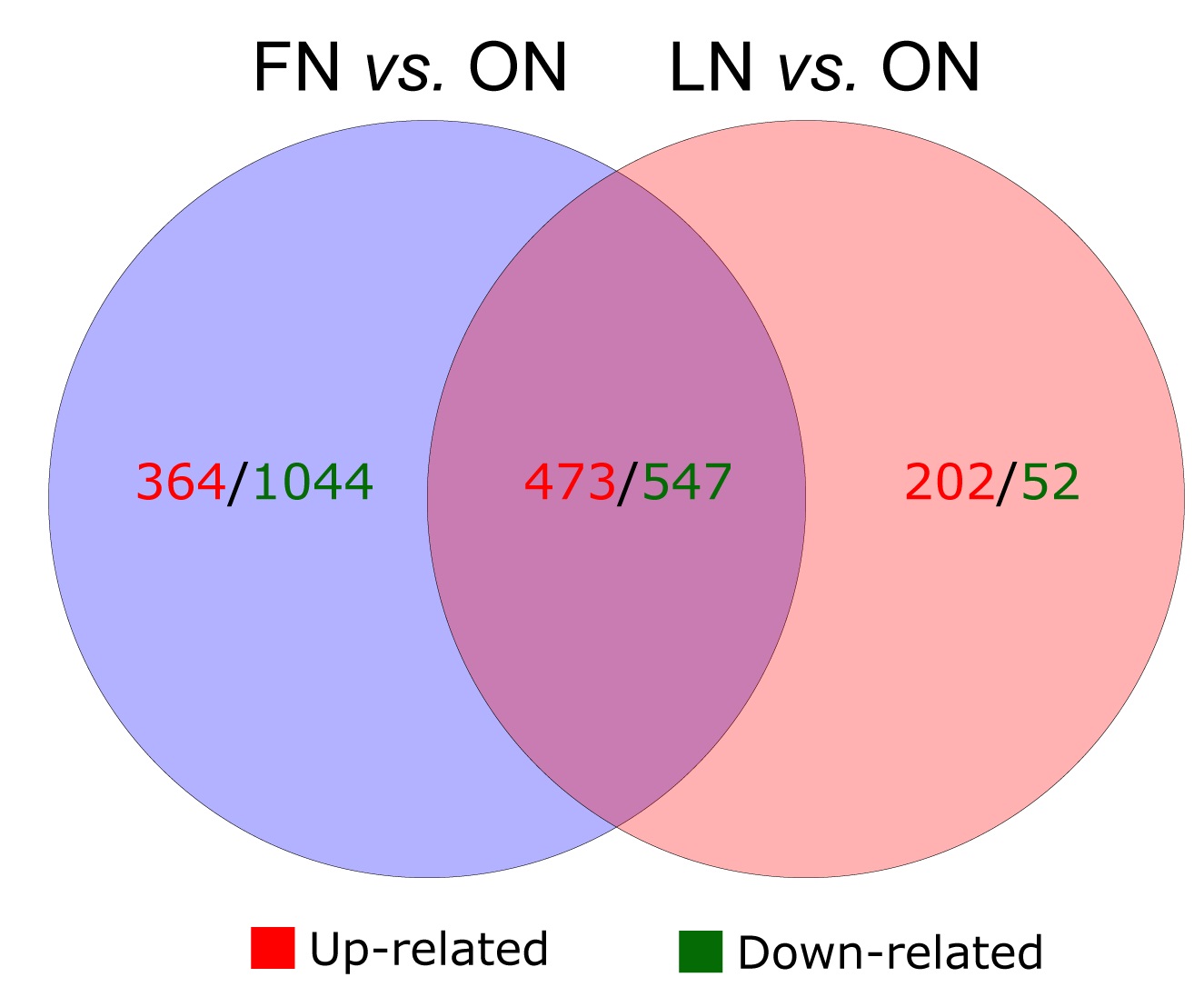

Supplement: Figure S4 — Common differentially expressed genes (DEGs) and their expression profile between free- (FN) vs. optimal- (ON) nitrate and low-nitrate (LN) vs. ON comparisons. [file Image4.JPEG]

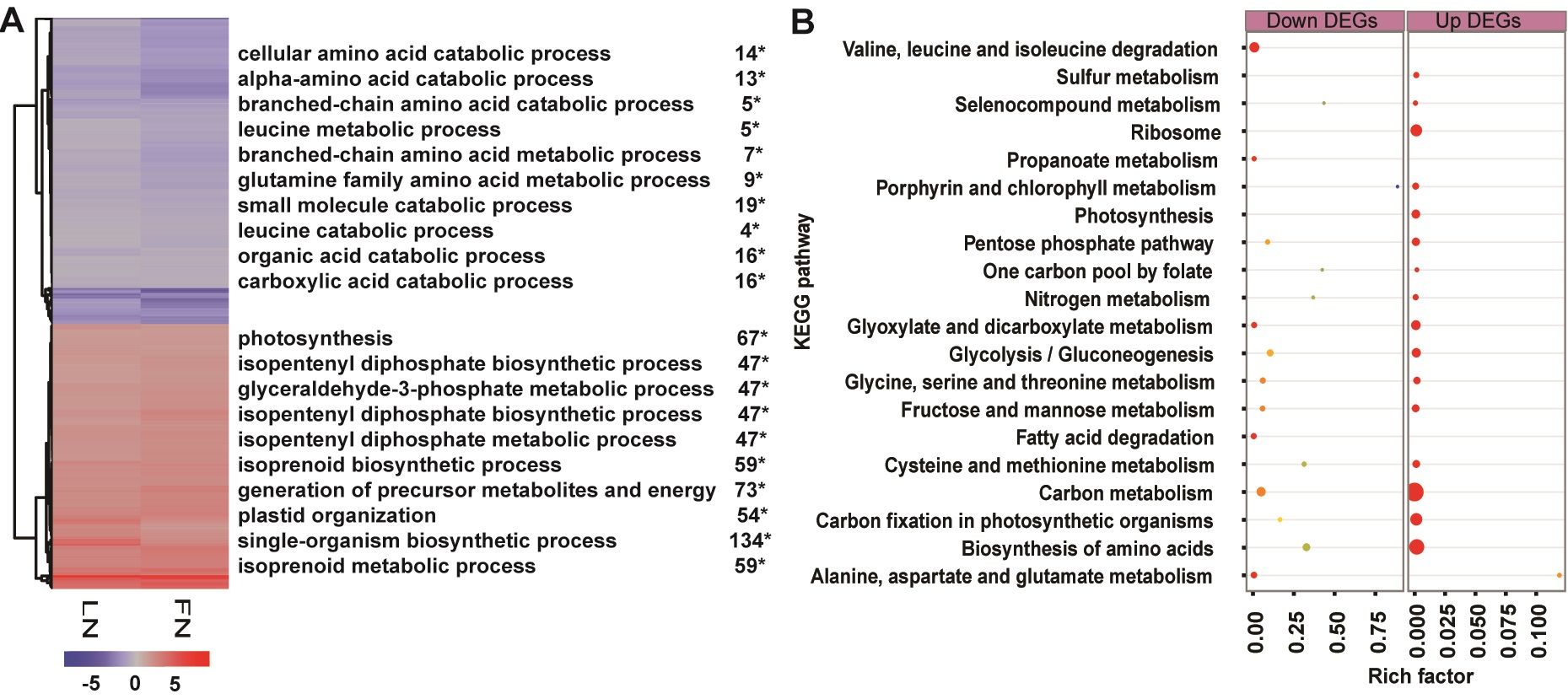

Supplement: Figure S5 — Functional annotation and enrichment analysis of common differentially expressed genes (DEGs) between free- (FN) vs. optimal- (ON) nitrate and low-nitrate (LN) vs. ON comparisons. (A) Clustering and heat maps of common DEGs based on the expression profiles. (B) KEGG pathway analysis of common DEGs. [file Image5.JPEG]

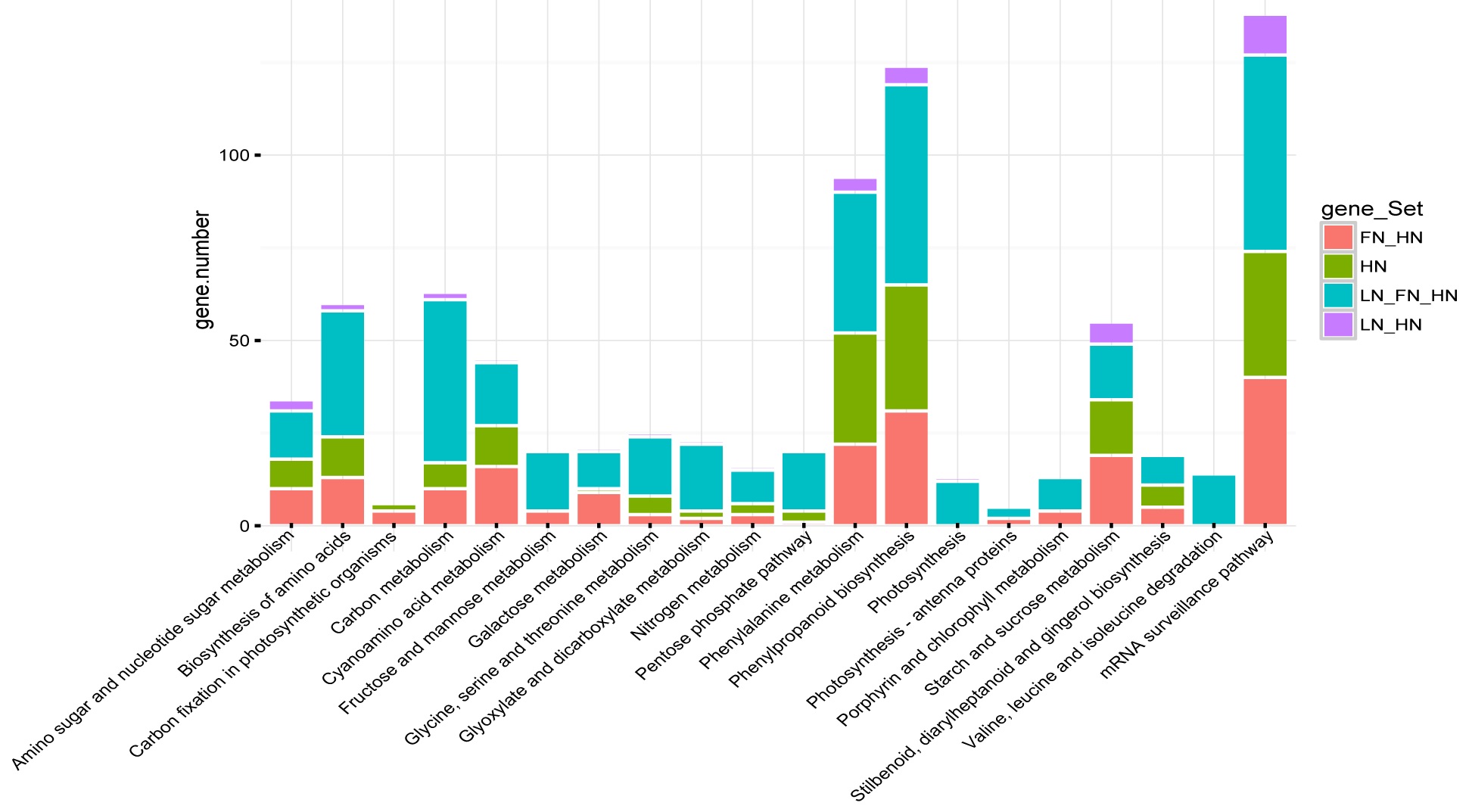

Supplement: Figure S6 — Cross-comparison of pathway annotation of differentially expressed genes (DEGs) in response to N stress. [file Image6.JPEG]
